# Dangerous liaisons: molecular basis for a syndemic relationship between Kaposi’s sarcoma and *P. falciparum* malaria

**DOI:** 10.3389/fmicb.2013.00035

**Published:** 2013-03-12

**Authors:** Katelyn L. Conant, Johnan A. R. Kaleeba

**Affiliations:** Department of Microbiology and Immunology, Uniformed Services University of the Health SciencesBethesda, MD, USA

**Keywords:** Kaposi’s sarcoma, HHV-8, malaria, *Pf*EMP-1, CD36, Basigin/CD147, *Pf*Rh5

## Abstract

The most severe manifestations of malaria (caused by *Plasmodium falciparum*) occur as a direct result of parasitemia following invasion of erythrocytes by post-liver blood-stage merozoites, and during subsequent cyto-adherence of infected erythrocytes to the vascular endothelium. However, the disproportionate epidemiologic clustering of severe malaria with aggressive forms of endemic diseases such as Kaposi’s sarcoma (KS), a neoplasm that is etiologically linked to infection with KS-associated herpesvirus (KSHV), underscores the significance of previously unexplored co-pathogenetic interactions that have the potential to modify the overall disease burden in co-infected individuals. Based on recent studies of the mechanisms that *P. falciparum* and KSHV have evolved to interact with their mutual human host, several new perspectives are emerging that highlight a surprising convergence of biological themes potentially underlying their associated co-morbidities. Against this background, ongoing studies are rapidly constructing a fascinating new paradigm in which the major host receptors that control parasite invasion (Basigin/CD147) and cyto-adherence (CD36) are, surprisingly, also important targets for exploitation by KSHV. In this article, we consider the major pathobiological implications of the co-option of Basigin/CD147 and CD36 signaling pathways by both *P. falciparum* and KSHV, not only as essential host factors for parasite persistence but also as important mediators of the pro-angiogenic phenotype within the virus-infected endothelial microenvironment. Consequently, the triangulation of interactions between *P. falciparum*, KSHV, and their mutual human host articulates a syndemic relationship that points to a conceptual framework for prevalence of aggressive forms of KS in malaria-endemic areas, with implications for the possibility of dual-use therapies against these debilitating infections in resource-limited parts of the world.

## INTRODUCTION

*Plasmodium falciparum* (*Pf*) malaria is one of the world’s leading health challenges, and at least two million people, mainly children below the age of 5 years, die each year from clinical complications of the disease (Snow et al., 2005). The most severe manifestations of *Pf* malaria occur as a direct result of parasitemia following invasion of erythrocytes by post-liver blood-stage merozoites, and during subsequent cyto-adherence of parasitized red blood cells (pRBCs) to the vascular endothelium and other host cells and tissues. Erythrocyte invasion is executed by a family of *Pf* reticulocyte-binding-like homolog (*Pf*Rh) ligands displayed on the surface of merozoites. Among at least five known members of this family, *Pf*Rh5 (Rodriguez et al., 2008; Lopaticki et al., 2011; Tham et al., 2012), which is indispensable for merozoite growth in cultures, is essential for invasion by all *Pf* strains (Baum et al., 2009; Lopaticki et al., 2011). The cognate erythrocyte receptor for *Pf*Rh5 was recently identified as CD147 [also known as Basigin (BSG), extracellular matrix metalloproteinase inducer (EMMPRIN), and leukocyte activation antigen, M6 (hereafter CD147); Crosnier et al., 2011]. This discovery is notable for the fact that the CD147/*Pf*Rh5 pair is essential for invasion of all laboratory-adapted and field strains of *Pf*, a cross-strain dependency that reveals opportunities for new anti-malarial therapies based on targeting this receptor/ligand interaction. Following invasion, extensive replication within infected human red blood cells (RBCs) results in surface expression of the multi-domain *Pf* erythrocyte membrane protein-1 (*Pf*EMP-1) family of genes (Baruch et al., 1995), including *Pf*EMP-1 that serves as a platform for sequestration of pRBC from the blood circulation by adhering to endothelial and other host cells and tissues (Berendt, 1993).

**FIGURE 1:**
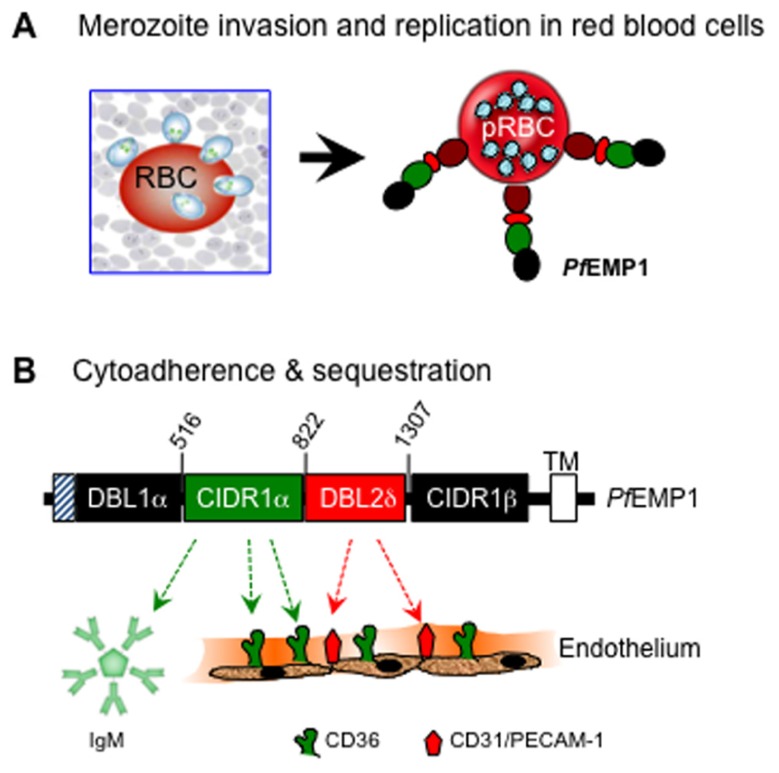
**Basic interactions of *P. falciparum* with human erythrocytes during invasion **(A)** and with blood capillary endothelium during cyto-adherence **(B)**.** Upon exit from the liver, invasion of red blood cells by the blood-stage merozoites leads to replication and subsequent surface expression of the multi-domain *Pf*EMP-1 molecule that mediates cyto-adherence via the binding activities of DBL1, CIDR1, and DBL2 domains of *Pf*EMP-1 with various host adhesion receptors. Shown here is a region of *Pf*EMP-1 encoded by FBR3S1.2-*var1* (Chen et al., 2000). The CIDR1 domain primarily binds to CD36 and to members of the immunoglobulin superfamily, including IgM and CD31/PECAM (platelet endothelial cell adhesion molecule), whereas the DBL2 domain binds mainly to CD31/PECAM-1.

The cyto-adhesive property of infected red blood cells to the microvasculature and sequestration within vital organ systems is an important survival strategy that allows the parasite to escape immune-mediated destruction (Urban et al., 1999). Cyto-adherence is mediated by an orchestrated set of interactions between specific regions within the ectodomain of *Pf*EMP-1 [notable among them being the cysteine-rich interdomain region (CIDR1α)], with a variety of host molecules on the surface of capillary endothelial cells. A well-characterized host receptor that mediates cyto-adherence of most *Pf* isolates to the peripheral vasculature is human CD36 (Ockenhouse et al., 1989), although other cell adhesion molecules are also involved in execution of strain and tissue-specific cyto-adhesive events (Ockenhouse et al., 1991; Baruch et al., 1997; **Figure 1**).

Recent studies have revealed that while erythrocyte invasion and cyto-adherence represent essential evolutionary strategies for parasite growth, survival, and persistence, they are also invariably associated with alteration of cellular physiology, which in turn may contribute directly to the defining clinical manifestations of *Pf* infection (Trossaert et al., 1991; Fried and Duffy, 1998). However, less examined is the provocative hypothesis that malarial disease may not be solely attributable to complications associated with the various stages of the *Pf* lifecycle alone; rather, the sequelae of illnesses associated with *Pf* malaria is likely to be the collective manifestation of a multitude of complex interactions between *Pf*, other co-pathogenic infections, and the human host. In this article, we highlight emerging evidence supporting the proposition that the signaling pathways anchored by Basigin/CD147 and CD36, two of the known host receptors that control *Pf* invasion and cyto-adherence, respectively, are also targets for functional subversion by Kaposi’s sarcoma (KS)-associated herpesvirus (KSHV), an inherently persistent cancer-associated herpesvirus that is prevalent in malaria-endemic regions. We discuss a number of surprising nodes of pathogenetic interface between *Pf* and KSHV in this context, and evaluate the major implications of the apparent co-option, by both *Pf* and KSHV, of CD147 and CD36 signaling pathways as a means to promote *Pf* persistence on one hand, and virus-induced regulation of the angiogenic phenotype, on the other. We then provide a synthesis of how the triangulation of interactions between *Pf*, KSHV, and their mutual human host represents the basis for a venerable syndemic relationship that may explain the co-incidence of aggressive forms of KS in malaria-endemic regions.

## THE MULTI-FACTORIAL ETIOLOGY OF KAPOSI’S SARCOMA

The most important pathological manifestation of KSHV infection is KS, a multifocal and highly angiogenic mesenchymal neoplasm characterized by profound inflammation and angio-proliferative expansion of spindle cells believed to be of endothelial origin (Bubman and Cesarman, 2003). KS occurs in at least four epidemiological forms, each with its own distinguishing clinical disposition determined by age, sex, geographical location, socio-economic status, previous exposure to parasitic infections, co-infection with the human immunodeficiency virus (HIV), and the extent of acquired or iatrogenic immunosuppression (Bubman and Cesarman, 2003; Dourmishev et al., 2003; Haverkos, 2008): thus, *iatrogenic* KS is mostly associated with organ (especially renal) transplantation and is mostly seen as localized skin lesions among people from areas where KSHV is endemic. *Epidemic* HIV/AIDS-associated KS is more commonly seen among HIV-infected individuals, while *classical* KS (cKS) manifests among older men of Mediterranean origin as red to purple skin plaques or nodules primarily on the lower extremities. *Endemic* KS (eKS), which is strikingly similar to cKS in its clinical disposition, is highly prevalent in East and Central Africa, where it affects children and young adults as a cutaneous disease invading soft tissue and bone, or as a fulminant lymphadenopathy that can rapidly disseminate to visceral organs (Hengge et al., 2002a,b). eKS is currently the most common cancer in adult East and Central African men and follows only cervical and breast cancer in adult women (Bassett et al., 1995; Wabinga et al., 2000; Casper, 2006). An important distinction is that whereas iatrogenic and AIDS-related KS are invariably associated with an immunosuppressed state, cKS and eKS are generally not (Kestens et al., 1985), implying that the development and/or propagation of the latter two types of KS (i.e., cKS and eKS) may be controlled by unique, geographically restricted co-factors unrelated to HIV or drug-induced immune suppression (Pyakurel et al., 2007).

Moreover, eKS in its most severe manifestation affects young, immunocompetent individuals whereas cKS afflicts older men who, beyond age-related senescence, do not display evidence of overt immune dysregulation, implying that the development and/or propagation of these forms of the lesion may be controlled by unique, geographically restricted co-factors unrelated to HIV or drug-induced immune suppression (Pyakurel et al., 2007).

A number of non-competing hypotheses have been proposed to explain the contribution of socio-economic, behavioral, and environmental co-factors to the histogenesis of eKS and cKS, the two types of the lesion not strictly dependent on HIV or iatrogenic immunosuppression. For example, clinical studies have revealed that eKS displays a notable predilection for the feet and legs of rural peasants and cultivators living in highland areas, thus inspiring the volcanic soil hypothesis first proposed by Ziegler (1993) and subsequently supported by additional epidemiologic studies (Montesu et al., 1995; Montella et al., 1996, 1997, 2000; Goedert et al., 2002). This hypothesis proposes that walking barefoot allows soil-borne aluminosilicates, iron oxides, and other clay minerals to be taken up through sweat glands and abrasions by resident macrophages, dermal microvascular endothelial cells, and by the lymphatic system. The resulting chronic lymphatic irritation, inflammation, and immune suppression could in turn support primary infection through these portals, and/or reactivation of latent KSHV within the epidermal aspects of skin. Lin and colleagues also recently suggested that exposure to soil and water constituted an additional risk factor for KS development by promoting parasitic infections that could either reduce local immune reactivity or induce a condition of inflammation necessary for KS development as well (Lin et al., 2008). Alternatively, the finding that some natural products of plants indigenous to the areas with eKS could reactivate KSHV (Whitby et al., 2007) inspired the “oncoweed” hypothesis of KS development. Subsequently, Ruocco et al. (2011) noted that quinine, an anti-malarial drug used extensively as one of the mainstays for treatment of malaria in immunocompetent children, may trigger KS development via its immunosuppressive effects that could support virus reactivation. It is therefore noteworthy that although available evidence is not sufficient to explain the distinctive geographical distribution of KS, the four known types of the lesion have a multi-factorial etiology, with the unifying theme being that the potential co-factors in each case operate at the level of the molecular mechanisms that control KSHV infection, dissemination, and the balance between virus replication and establishment of latency. This is a relevant link given the fact that a sustained state of low level lytic replication may be important for histogenesis and probably propagation of the KS lesion (Grundhoff and Ganem, 2004).

## CO-PATHOGENIC MECHANISMS OF KS AND MALARIA: A HYPOTHESIS

Like Burkitt’s lymphoma, KS is one of the most prevalent childhood cancers in malaria-endemic areas. The incidence of KS displays a considerable degree of geographic variation that mirrors the prevalence of its causative agent, and may depend on etiologic mechanisms that are controlled by geographically restricted co-factors, including malaria endemicity (Ziegler et al., 1984, 2003; Ascoli et al., 2001, 2006a,b; Coluzzi et al., 2003, 2004b; Wakeham et al., 2011). For example, KS incidence is particularly high in sub-Saharan Africa, a region with one of the highest rates of malaria deaths (Snow et al., 2005). Epidemiological studies have also shown a disproportionately high incidence of KS among elderly men in Greece, Turkey, Israel, and in Italy where the greatest number of recorded cases are in the formerly malaria-endemic provinces of Sardinia, Sicily (Vitale et al., 2001), and in the Po valley (Ascoli et al., 2001). Such overlapping geographic clustering of *Pf* malaria with eKS or cKS has inspired the co-pathogenesis hypothesis, which proposes that a previous or ongoing exposure to KSHV in a setting of underlying parasite persistence (or vice versa) may result in molecular interactions between *Pf* and KSHV that could modify the overall disease burden exerted by both pathogens at a micro level. Based on this attribution, we refer to the putative co-morbidity of KS and *Pf* malaria (in settings for which the evidence for this linkage is strong), as representing an example of a classic syndemic relationship articulated by the display of co-incident clustering within defined geographic regions.

In spite of evidence from case–control studies that show a disproportionately high KS prevalence in areas of currently or previously high malaria endemicity, a molecular link between KS pathogenesis and *Pf* malaria has not been rigorously examined at a micro level, and the correlation is even more difficult to establish at a population level because of the indeterminate nature of the mechanisms by which malaria might influence KS pathogenesis outside the known clusters of endemic disease co-incidence. On one hand, the role of malaria as a co-factor for eKS has been hypothesized based on the potential of the *Anopheles* mosquito vector to contribute to person-to-person spread of KSHV, and to establish an immunosuppressed state at the site of the mosquito bite, which would then create a local permissive environment for KSHV infection (Coluzzi et al., 2003; Ascoli et al., 2006a,b,c). However, we propose an alternative model based on the provocative hypothesis that both the malaria parasite and KSHV exert bidirectional influences upon each other that operate at a much more complex level beyond the permissive benefits of immune suppression or the modifying effects of occupational, socio-economic, or environmental co-factors.

As a conceptual basis for our hypothesis, consideration of some of the known molecular controls that regulate the KSHV life cycle reveals a number of insights into the potential pathobiological link between KS and malaria. Like Epstein–Barr virus (EBV) and related herpesviruses, KSHV establishes both lytic and long-term latent infections, the balance between which determines the timing and intensity of pathologic outcomes in specific organ systems. Interestingly, both KSHV and EBV are highly prevalent in malaria-endemic areas and also share many features in their life cycles, including the manner in which they regulate the molecular switch between latency and lytic replication. For example, expression of the major KSHV replication and transcription activator (RTA) is essential for virus replication and dissemination, and is dependent on activation of p38 and extracellular signal-related kinase 1/2 (ERK1/2) mitogen-activated protein kinase (MAPK; Xie et al., 2008). KSHV RTA is the genetic and functional homolog of EBV RTA/BRLF1 (Bam HI fragment Z rightward open-reading frame 1) which, in concert with Zebra Transcriptional Activator/BamHI fragment Z rightward open-reading frame 1 (ZTA/BZLF1), can initiate EBV lytic cycle (Staudt and Dittmer, 2007). KSHV RTA positively regulates immediate and delayed early promoters as well as its own promoter by interacting with cellular transcription factors such as activator protein-1 (AP-1), octamer binding transcription factor 1 (Oct-1), recombination signal-binding protein Jkappa (RBP-Jκ), and CCAAT/enhancer binding protein alpha (C/EBPα; Staudt and Dittmer, 2007); in this way, RTA is susceptible to a variety of signal transduction pathways that are likely to activate its promoter. In fact, KSHV RTA expression (and therefore lytic replication) can be induced *in vitro* using a variety of chemical compounds such as ionomycin (a calcium ionophore), sodium *n*-butyrate (NaB, a histone deacetylase inhibitor) and phorbol esters such as 12-*O*-tetradecanoylphorbol-13-acetate (TPA), all of which can also be used to induce EBV lytic cycle (Luka et al., 1979; Renne et al., 1996; Zhu et al., 1999; Gao et al., 2001).

Given the shared genetic and biological properties of EBV and KSHV, it is not surprising that the two viruses can co-exist as latent episomes in certain peripheral effusion lymphoma-derived cell lines (Horenstein et al., 1997). They also display similarities not only in the mechanism of induction of the lytic cycle but also in the distribution of endemic cancers associated with them in regions of high malaria endemicity, which perhaps reflects the contribution of malaria as a common co-factor in the pathogenesis of cancers associated with these viruses. For EBV, the epidemiological association between malaria and African endemic Burkitt’s lymphoma (eBL) is well established (Moormann et al., 2011), although the molecular mechanisms by which malaria modifies eBL and other pathobiological outcomes of EBV infection remain a matter of intense investigation (Rochford et al., 2005). Interestingly, eBL is a B cell lymphoma propagated by a deregulation in the *c-myc* oncogene as a result of chromosomal translocation, and although EBV is a necessary etiological agent for eBL, it is clearly not sufficient in absence of other essential co-factors. It is also worth noting that whereas nearly all African children in endemic areas suffer from several malaria episodes as a result of chronic exposure to *Pf*, only a fraction of them show signs of severe, life-threatening forms of the disease, implying that the biological processes underlying the progression of infection to disease are much more complex. Interestingly, EBV is ubiquitous in the general human population, and in children living in malaria endemic areas, primary infection can occur within a few months of birth (Piriou et al., 2012), and may seroconvert within 3 years after primary infection, followed by a tightly orchestrated viral latency state in memory B cells that reflects the balance between viral replication and host immune control. Emerging new evidence now suggests that the exit from latency to the viraemic state that supports eBL development may be impacted at a molecular level by the replicative blood-stage form of the parasite life cycle. Thus, Chene et al. (2007) recently demonstrated that interactions between the CIDR1α domain of *Pf*EMP-1 on the surface of pRBCs, with a cognate surface receptor(s) expressed on the surface of EBV-infected human memory B cells, stimulates B cell proliferation (as previously shown; Donati et al., 2004) and reactivates EBV not only from the chronically infected Akata cell line but also from latently infected B cells isolated from the peripheral blood and tonsils of healthy EBV carriers (Chene et al., 2007). The mechanism by which conjugation of *Pf*EMP-1-expressing pRBC with EBV-infected B cells triggers virus replication in this model remains to be elucidated, but the consequences of this interaction can be readily appreciated as providing a possible explanation for the increased EBV viral load that constitutes risk for eBL among children living in areas of high malaria endemicity.

**FIGURE 2:**
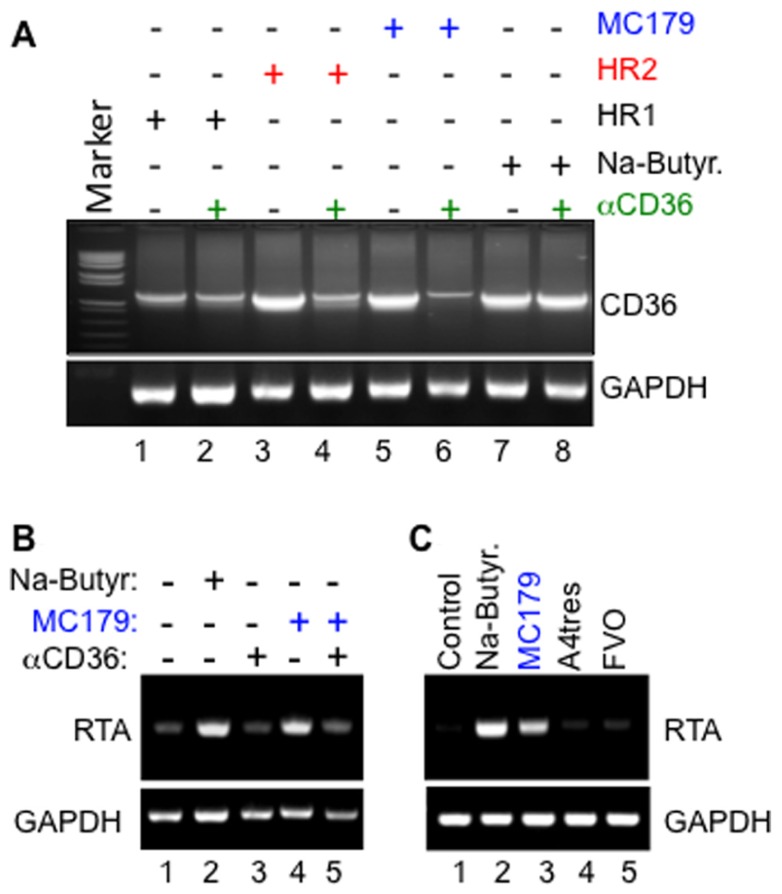
**(A)** Upregulation of CD36 by both the *Pf*EMP-1-CIDR1α-derived MC179 peptide (lanes 5 and 6) and by KSHV gB-derived heptad peptide ligands HR2 (lanes 3 and 4), and the less potent HR1 (lanes 1 and 2) are both blocked by a monoclonal anti-CD36 antibody. Note that anti-CD36 did not block control upregulation of CD36 in response to treatment with sodium butyrate (NaB; lanes 7 and 8). **(B)** Activation of KSHV RTA by MC179 occurs via a CD36-dependent mechanism that can be blocked by anti-CD36 antibody FA6-152. *Methodology*: briefly, melanoma-derived Mel1700 cells were seeded in a six-well plate and either left untreated or pre-incubated with 10 μg/ml of anti-CD36 monoclonal antibody clone FA6-152 for 25 min. at 25°C. After washing to remove excess antibody, cells were incubated with either 100 μg/ml of KSHV gB-derived HR1, 200 μg/ml HR2 peptide, 100 μg/ml recombinant MC179, or 2 mM sodium butyrate (NaB). Forty-eight hours after treatment, total RNA was isolated and used as template in semi-quantitative RT-PCR with primers to an internal fragment of human CD36, viral RTA, or human glyceraldehyde 3-phosphate dehydrogenase (GAPDH) loading control. **(C)** Specific activation of the KSHV lytic switch protein, RTA, by the *Pf*EMP-1-CIDR1α-derived peptide MC179 from *Pf* Malayan Camp strain, but not by peptides derived from CIDR1α domains of A4tres (which binds ICAM-1) or the Vietnam Oak Knoll strain (FVO).

Remarkably, we have also discovered that cross-linking of CD36 on the surface of KSHV-infected cells with MC179, a recombinant peptide derived from the CIDR1α domain of *Pf*EMP-1 that normally interacts with CD36 to mediate cyto-adherence (Ockenhouse et al., 1989, 1991; Baruch et al., 1997), not only upregulated CD36 expression (**Figure 2A**) but also reactivated the virus from latency through transcriptional activation of KSHV RTA (**Figure 2B**), and that the molecular mechanisms that control this process overlap with those that putatively regulate *Pf*EMP-1-dependent EBV reactivation from latently infected cells (Chene et al., 2007). Remarkably, structural mimics of MC179, including the two helical heptad repeats (HR1 and HR2) derived from KSHV entry glycoprotein B (gB) also upregulated CD36 expression and further induced virus reactivation in a CD36-dependent manner. Indeed, like MC179, the effect of these helical peptides could be blocked by a monoclonal antibody to CD36 (**Figures 2A,B**), suggesting that they bind a region on the exoplasmic face of CD36 that overlaps with MC179. It is also insightful that the KSHV lytic cycle was activated only by the *Pf*EMP-1-CIDR1α-derived peptide from *Pf* Malayan Camp strain, but not by peptides derived from the Vietnam Oak Knoll (FVO) or A4tres strain [that preferentially binds intercellular adhesion molecule 1 (ICAM-1) and not CD36] (**Figure 2C**), demonstrating that reactivation of KSHV by the parasite ligand displays some degree of strain specificity for *Pf* that is associated with severe malaria in Africa.

**FIGURE 3:**
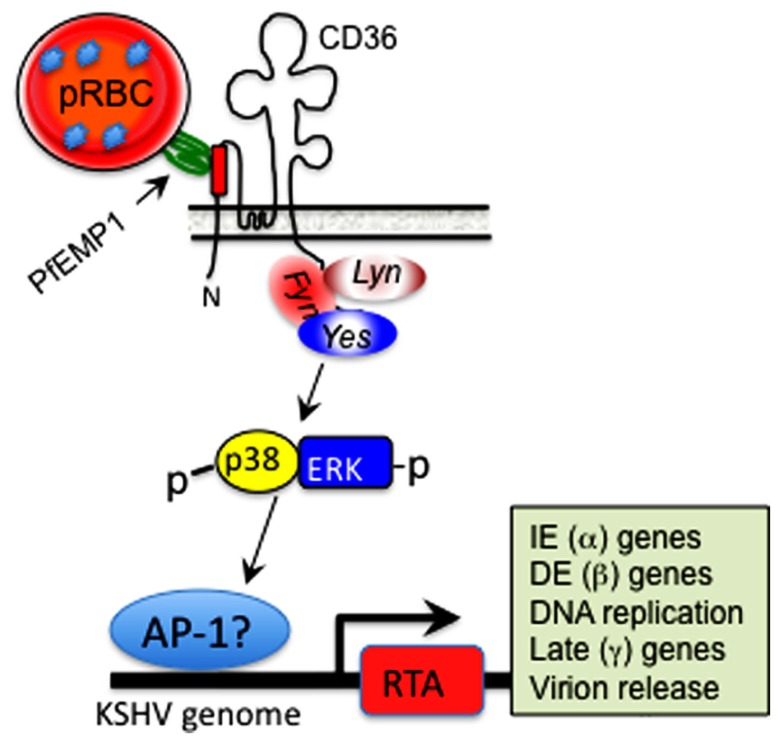
**Hypothetical model of CD36-dependent RTA activation**. A motif displayed by *Pf*EMP-1 on the surface of parasitized erythrocytes interacts with its cognate epitope within the ectodomain of CD36 on the surface of microvascular endothelial cells. This interaction activates one or more of the *Src*-like kinases, which in turn initiate a phosphorylation cascade that results in p38 and ERK/MAPK activation. This process culminates in activation of a cellular transcription factor, likely AP-1 (dimer of *c-jun* and *c-fos*), which translocates into the nucleus and stimulates KSHV RTA-dependent transcription of viral lytic cycle genes, starting with immediate early (IE), which then activate delayed early (DE), followed by late structural genes involved in assembly of an infectious virus particle.

Since CD36 mediates parasite persistence by mediating cyto-adherence and sequestration of parasitized erythrocytes away from immune surveillance, the ability of MC179 to stimulate KSHV reactivation is significant, as it supports a model whereby *in vivo* cross-linking of CD36 on the surface of KSHV-infected cells, either by its natural ligand(s) or upon conjugation with the CIDR1α domain of *Pf*EMP-1 expressed on the surface of parasitized erythrocytes (as illustrated in **Figure 3**), represents a previously unrecognized mechanism by which KSHV lytic replication could be induced in the context of a *Pf* malaria co-infection. This model provides many new opportunities for experimental examination of the ability of parasite-derived antigens to reactivate KSHV during cyto-adherence on the surface of infected blood endothelial cells and within tissues and organs: (i) Biochemical analysis of structure/function relationships that control interactions of CIDR1α (and its structural analogs) with CD36, should reveal potential targets for small-molecule inhibition of parasite-induced sequestration or reactivation of KSHV in co-infected individuals. (ii) Elucidation of the signaling mechanisms that regulate CD36-dependent RTA activation will require multi-pronged approaches that employ dominant negative versions of the *Src*-like kinases such as *yes*, *fyn*, and *lyn* that control down-stream signaling events initiated by CD36 ligation (see **Figure 3**). (iii) Deletional mutagenesis, domain-swapping, functional complementation, and loss-of-function analysis of genetic mimics of the recently identified CD36 polymorphisms that lack the signaling motif (Aitman et al., 2000; Gelhaus et al., 2001; Chilongola et al., 2009; Fry et al., 2009) should elucidate the correlates of upstream signaling networks required for CD36-dependent activation of the KSHV lytic cycle.

## CD36 AND THE *Pf* LIFECYCLE: IMPLICATIONS FOR KSHV PATHOGENESIS IN THE CO-INFECTED HOST

CD36 is a class II glycoprotein involved in multiple physiological functions including cell adhesion, fatty acid uptake, non-opsonic phagocytosis, and angiogenesis (McGilvray et al., 2000; Podrez et al., 2007; Silverstein and Febbraio, 2007, 2009). The CD36 structure consists of a large extracellular loop and two short cytoplasmic tails at the N- and C-termini (Silverstein and Febbraio, 2009). The C-terminal tail is involved in signal transduction via association with *Src*-like kinases, whereas the extracellular domain contains binding sites for thrombospondin-1 (TSP-1), a potent natural inhibitor of angiogenesis (Bagavandoss and Wilks, 1990; Good et al., 1990; Tolsma et al., 1993), and a variety of other ligands including the CIDR1α domain of *Pf*EMP-1 (Ockenhouse et al., 1989). Although CD36 has never been directly associated with KSHV pathogenesis, some of its pleiotropic functions are consistent with its potential contribution to the basic pathobiology of KSHV. For example, CD36 is often expressed in association with signaling structures such as lipid rafts that also contain host receptors for KSHV entry, including integrins and the KSHV fusion receptor complex xCT/CD98 (Akula et al., 2002; Kaleeba and Berger, 2006; Veettil et al., 2006). Since CD36-mediated signaling may culminate in activation of p38 and ERK MAPK pathways that overlap with one or more pathways necessary for KSHV RTA-dependent transcription of viral gene expression (Yipp et al., 2003; Cohen et al., 2006; Xie et al., 2008), our data suggests that CIDR1α-dependent RTA activation may occur via by signals transduced through CD36 (**Figure 3**). However, our model also raises many significant questions: (a) Does CIDR1α-induced KSHV replication involve mechanisms that overlap with, or distinct from those that support CIDR1α-induced EBV replication (Chene et al., 2007)? (b) Does it require the traditional CD36-regulated recruitment of *Src*-like kinases and subsequent phosphorylation of p38 and ERK MAPK? (c) Does it directly result in downstream activation of a specific cellular transcription factor such as AP-1 that is known to activate KSHV RTA (or EBV ZTA) promoter(s) (e.g., see **Figure 3**)? (d) Is it strictly dependent on interactions of CIDR1α with a distinct epitope on CD36, or does it overlap with, and is therefore also inducible by, other CD36 ligands such as TSP-1? Investigation of the role of TSP-1 in this context is relevant, given that TSP-1 is present in high concentrations in human saliva (Crombie et al., 1998, 2001; Shugars, 1999) that generally contains significant levels of KSHV virions shed from the underlying oral zones of carriers (Pauk et al., 2000; Coluzzi et al., 2004a; Chene et al., 2007; Hadinoto et al., 2009). Critical new experiments that address these derivative questions represent an exciting area of research into the molecular basis for the emerging new paradigm of co-pathogenesis. It is also anticipated that isolation of polymorphisms in CD36 and other human genes that control host interactions with *Pf*, EBV and KSHV may open up additional opportunities for population-level studies aimed at explaining the overlapping distribution of KS, Burkitt’s lymphoma, and malaria in areas where these diseases display coincident endemicity.

## VIRAL SUBVERSION OF THE CD36 SIGNALING PATHWAY: IMPLICATIONS FOR PARASITE SEQUESTRATION AND ANGIOGENESIS

**FIGURE 4:**
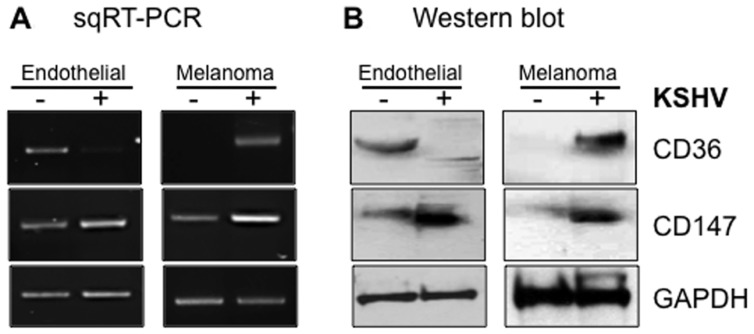
**Cell type-specific outcomes of KSHV infection in skin versus endothelial cells with respect to CD36 and CD147 expression**. Semi-quantitative RT-PCR **(A)** and Western blot **(B)** analysis of CD147 and CD36 expression in uninfected (-) versus infected (+) endothelial (lymphatic) or melanoma-derived Mel1700 cells. KSHV upregulates both CD36 and CD147 in melanoma-derived cells, whereas in lymphatic and telomerase-immortalized mixed dermal microvascular and brain endothelial cells, KSHV upregulates CD147 (confirming a recent study; Qin et al., 2010) but downregulates expression of CD36 (and its angiostatic ligand, TSP-1; data not shown); *in vivo*, these dichotomous effects are consistent with promotion of angiogenesis, invasion, and tumor metastasis in disease-relevant cell types.

Angiogenesis, defined as the development of new blood vessels, is necessary for growth and proliferation of vascular tumors like KS, the extent of which is controlled by the balance between pro-angiogenic and angiostatic elements of the human hemostatic system. One of the regulatory components of this system is TSP-1, the angiostatic CD36 ligand known to inhibit endothelial cell proliferation, migration, and tube formation (Iruela-Arispe et al., 1991). Remarkably, KSHV upregulates CD36 in melanoma-derived cell lines but downregulates the protein in endothelial cells both at mRNA and protein levels (**Figure 4**). The mechanism(s) by which KSHV accomplishes these dichotomous, cell type-specific effects are not fully understood, but one study recently demonstrated that KSHV-encoded microRNAs can directly target TSP-1 mRNA for degradation (Samols et al., 2007), ostensibly to promote an angiogenic growth state of infected cells via attenuation of the angiostatic effects resulting from interactions between TSP-1 and CD36. Since the angiostatic signal is associated with viral lytic replication while the angiogenic phenotype is linked to the latency phase, virus regulation of CD36 expression and signaling in infected cells implies that the correlates of KSHV latency are contextually linked to the angiogenic phenotype in disease-relevant cells. They also provide a potential explanation for our recent findings that the KSHV latency program is inefficient in melanoma cells from which the virus undergoes robust spontaneous replication, as opposed to endothelial cells in which the virus establishes a much tighter state of latency (Fontana et al., unpublished findings).

In addition to direct targeting of TSP-1, KSHV blunts CD36 signaling in endothelial cells by upregulating the endothelin (ET-1) system. ET-1 is a pro-angiogenic peptide secreted by the vascular endothelium and its deregulation is implicated in the pathogenesis of many malignancies (Nelson et al., 2003). ET-1 polypeptides and their cognate receptors are expressed in KS lesions (Nelson et al., 2003; Basilico et al., 2004), and ET-1 receptor blockade limited KS cell invasion in an *in vivo* tumor growth model (Rosano et al., 2003). The interplay between the pro-angiogenic effects of ET-1 and the pathophysiology of KS is also encountered in patients with complicated *Pf* malaria in which plasma concentrations of big ET-1, the precursor for bioactive ET-1, are elevated (Wenisch et al., 1996) as a direct result of cyto-adherence of pRBCs to human endothelial cells, independent of the parasite strain and regardless of the origin of endothelial cells (Basilico et al., 2004). There is, therefore, a significant degree of molecular crosstalk between *Pf* and KSHV at the level of ET-1 biology. Considered in a broader context, it is conceivable that by suppressing the angiostatic effects of CD36 signaling either by reducing CD36 expression, downregulation of TSP-1, or via upregulation of ET-1, KSHV could establish long-term persistence by establishing a state of limited CD36-dependent viral reactivation, or reduce CD36-dependent cyto-adherence and consequently limit the frequency of illnesses associated with this aspect of the parasite life cycle. Unfortunately, the latter outcome might be associated with an increase in the likelihood for parasite access to the extra-peripheral organs such as the brain, which may elevate the probability of cerebral malaria. Although measurement of these parameters *in vivo* is not trivial, a number of guiding principles can emerge from *in vitro* scrutiny of these molecular interactions based on experimental approaches that might predict their occurrence *in vivo*.

## CD36 POLYMORPHISMS DO NOT PROTECT FROM MALARIA: PERSPECTIVES ON SELECTION PRESSURE

It was recently discovered that many people of African origin harbor a high frequency of mutations and single-nucleotide polymorphisms (SNPs) that cause a deficiency in the CD36 gene, yet they still suffer from severe (particularly cerebral) malaria (Aitman et al., 2000; Gelhaus et al., 2001; Chilongola et al., 2009; Fry et al., 2009). The SNPs found in Kenya and Gambia introduce a premature stop codon that results in a truncated CD36 protein lacking the C-terminus and is therefore incompetent for signal transduction but can still bind its ligand(s), leading to the conclusion that mutations that cause CD36 deficiency may reduce CD36-mediated parasite sequestration in peripheral organs but they may not protect from severe cerebral malaria (Aitman et al., 2000) that is associated with cyto-adherence to brain endothelium via interactions of *Pf*EMP-1 with ICAM-1 but not CD36 (Ockenhouse et al., 1991). This level of linkage in which mutations in CD36 – a molecule that is important for parasite persistence – cause a deficiency that does not alter malaria pathogenesis, implies existence of selection pressures that may be induced or maintained in the population by an endemic infection (other than malaria) whose persistence is linked to this genetic output.

The fact that the mutations and their phenotypes occur at high frequency in malaria-endemic areas with a high prevalence of viruses associated with endemic cancers underscores a pathobiological paradigm whereby an inherently persistent tumor virus such as KSHV (or EBV) could provide the selective pressure for introduction or maintenance of such a mutation into the genetic registry of populations living in regions of high malaria endemicity. Given that *Pf*EMP-1 interactions with CD36 result in induction of the viral lytic cycle, such a virus-induced genetic output would conceivably be designed to promote virus escape from immune surveillance by limiting virus reactivation that might result from interactions between *Pf*EMP-1 and CD36 on latently infected cells. The impact of such a genetic influence, can only be measured against the host’s ability to restrict virus replication and dissemination, and it could be achieved by interrogating viral genomic variability or stability in a given population against a profile of polymorphisms within the genetic registries at the CD36 locus on a population basis. Availability of patient samples with known patho-status and disease severity from regions in which the distribution of malaria overlaps with the incidence of virus-associated endemic cancers would facilitate such a retrospective analysis.

## CD147 AND KSHV PATHOGENESIS

CD147 is a widely expressed, type I integral membrane receptor that belongs to the immunoglobulin (Ig) superfamily (Biswas et al., 1995). It is over-expressed in a variety of disseminated human solid cancers and is a major contributor to the malignant phenotype in a variety of human cancers (Riethdorf et al., 2006). Signaling events transduced through CD147 are associated with survival, metastasis, and invasion of a variety of cancer cells, mainly because it stimulates enhanced stromal release of multiple matrix metalloproteinases (MMPs) and vascular endothelial growth factor (VEGF), which are among the key mediators of angiogenesis and metastatic transition (Marieb et al., 2004; Tang et al., 2005; Bougatef et al., 2009; Kanekura and Chen, 2010). CD147 promotes hyaluronan synthesis (Marieb et al., 2004; Slomiany et al., 2009a), upregulates the Wnt/β-catenin signaling pathway (Sidhu et al., 2010), and is also involved in epithelial-to-mesenchymal transition (EMT; Wu et al., 2011). Given the pleiotropic function of CD147, the significance of CD147/*Pf*Rh5 interactions in parasite invasion unlocks new avenues for investigating the nodes of pathogenetic intersection between *Pf* malaria and other co-infecting agents such as KSHV that have evolved mechanisms to subvert the CD147 signaling pathway to promote their existential success.

**FIGURE 5:**
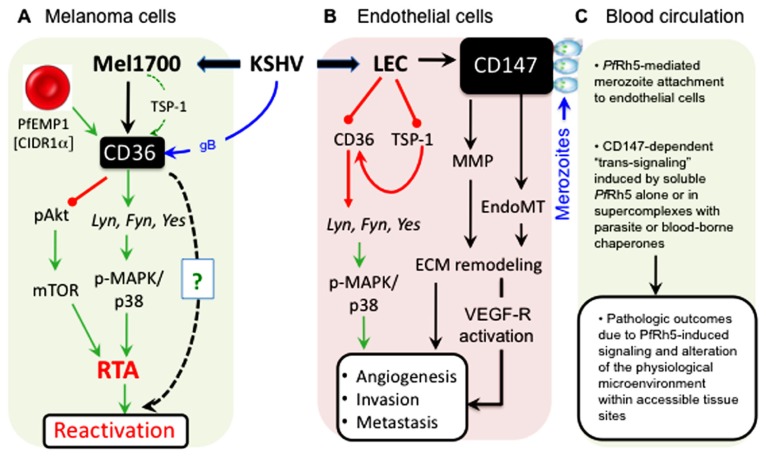
**Cell type-specific outcomes of KSHV interactions with CD36 and CD147 signaling pathways**. **(A)** In melanoma-derived cells (Mel1700), KSHV upregulates both CD36 (and its ligand, TSP-1) and CD147 (as shown in **Figure 4**). Binding of the *Pf*EMP-1 CIDR1α-derived peptide, MC179, to virus-upregulated CD36 prevents Akt phosphorylation while inducing RTA-dependent KSHV reactivation via the MAPK/p38 pathway. Remarkably, structural mimics of MC179, such as the helical heptad repeat (HR) regions derived from KSHV glycoprotein B (gB), can, like MC179, also induce virus reactivation in a CD36-dependent manner (as shown in **Figure 3**). **(B)** In lymphatic (LEC) and other endothelial cells such as mixed tolemerase-immortalized dermal microvascular and brain endothelial cells (tDMB), KSHV upregulates CD147 but unlike in Melanoma cells, the virus downregulates both CD36 and its angiostatic ligand, TSP-1; *in vivo*, these effects are likely to promote angiogenesis, invasion, and tumor metastasis. **(C)** As a key receptor for the merozoite invasion antigen, *Pf*Rh5, KSHV-induced upregulation of CD147 on the surface of infected endothelial cells increases the frequency of contacts between merozoite-bound or soluble *Pf*Rh5 and blood or dermal microvascular endothelial surfaces, resulting in induction of CD147-mediated signals that could alter the microenvironment and cause pathologic outcomes in a variety of physiological sites in the co-infected host.

Several layers of this pathogenetic intersection are revealed by new data on the manner in which both *Pf* and KSHV have evolved to exploit CD147 and endothelial cell biology:

a. Whereas endothelial cells play a prominent role as a platform for cyto-adherence of pRBCs, KSHV displays profound tropism for this cell lineage that represents the basis for origination of the hyper-proliferating spindle cells characteristically found in KS lesions (Bubman and Cesarman, 2003; Ganem, 2006).
b. Recent studies demonstrated compelling evidence that *de novo* KSHV infection of human endothelial cells as well as oral and fore-skin-derived fibroblasts results in upregulated expression of CD147 and that this effect is directly associated with various virological outcomes consistent with a pro-invasive, migratory, and pro-angiogenic phenotypes (Qin et al., 2010; Dai et al., 2012a,b). We have also found that KSHV upregulates CD147 not only in melanoma-derived cells but also in chronically infected lymphatic, microvascular, and brain endothelial cells (e.g., see **Figure 4**).
c. Two recent reports showed that KSHV promotes endothelial-to-mesenchymal transition (endo-MT) through activation of Notch-dependent signaling events that culminate in stimulation of an invasive phenotype analogous to that orchestrated by CD147 (Cheng et al., 2011; Gasperini et al., 2012). KSHV-induced endo-MT was dependent on the activity of membrane-type-1 MMP (MT1-MMP; Cheng et al., 2011), which is consistent with a role for CD147 in endo-MT since MT1-MMP is a CD147-stimulated endopeptidase involved in extracellular matrix remodeling. It is therefore conceivable that one of the mechanisms underlying KSHV-induced endo-MT may involve viral induction of MMP activity (Qian et al., 2007), likely through upregulation of CD147.
d. CD147 has been implicated in the entry processes of a number enveloped viruses including HIV-1 (Pushkarsky et al., 2001), measles virus (Watanabe et al., 2010), and severe acute respiratory syndrome coronavirus (SARS-CoV; Chen et al., 2005). Based on our recent studies, we also have reason to believe that CD147 may regulate KSHV entry as well, as an anti-human CD147 (neurothelin) antibody can potently block KSHV glycoprotein-mediated fusion, consistent with existence of CD147 in the membrane of KSHV-permissive cells as part of a molecular supercomplex (Guo et al., 2000; Yang et al., 2007; Gallagher et al., 2009) that includes host molecules implicated in virus entry, such as integrins (Akula et al., 2002) and the cystine transporter complex xCT/CD98hc (Xu and Hemler, 2005; Kaleeba and Berger, 2006). It is also noteworthy that, like CD36, CD147 also associates with the xCT/CD98hc complex and confers resistance to some chemotherapeutic drugs (Okuno et al., 2003; Yang et al., 2007; Zou et al., 2007). Interestingly, another independent study also demonstrated that the transport activity of xCT/CD98hc, perhaps in association with CD147, is a critical correlate of resistance to cisplatin (Huang et al., 2005; Singh et al., 2010; Chen et al., 2011; Riglar et al., 2011). For KSHV-infected individuals, one implication of these molecular associations could be that virus-induced upregulation of CD147 (as we and others have shown; Qin et al., 2010; Dai et al., 2012a,b), could stabilize CD147-containing multi-partite complexes and in turn potentiate their drug efflux functions, which could blunt the efficacy of chemotherapeutic strategies that might be used in the treatment of KS and other virus-associated malignancies. In support of this view, Qin et al. (2011) recently showed that the intrinsic resistance of KSHV-positive peripheral effusion lymphomas to the cytotoxic effects of paclitaxel and doxorubicin depends on orchestrated interactions of CD147 with lymphatic vessel endothelial receptor 1 (LYVE-1) and the homodimeric ATP-binding cassette (ABC)-G2/BCRP (breast cancer resistance protein) drug transporter that is highly expressed on the surface of primary effusion lymphoma (PEL)-derived cell lines.
e. Available evidence suggests that “outside-in” signaling may be required for the malaria parasite invasion (Singh et al., 2010; Chen et al., 2011; Riglar et al., 2011), but it remains to be determined if the structural framework that supports erythrocyte invasion through CD147 is distinct from, or overlaps with, the epitope on the CD147 ectodomain that senses signals transduced to endothelial and other cells that express this molecule. If they are the same, *Pf*Rh5-mediated conjugation of merozoites with CD147 on normal or infected endothelial cell surfaces should result in so-called parasite-to-host “trans-signaling” events that may lead to untoward pathologic outcomes unrelated to parasite invasion itself. Given that *Pf*Rh5 has neither a transmembrane domain nor a cytoplasmic tail, the probability of trans-signaling is likely to be high since a soluble form of *Pf*Rh5 [existing either as a monomer, as part of a bioavailable complex with another pathogen molecule such as *Pf*Ripr (Chen et al., 2011), or as bound to a host “handler”] could initiate CD147-dependent signaling outcomes that are likely to modify *Pf* malaria or KSHV infection (Marieb et al., 2004; Xu et al., 2007; Ruiz et al., 2008; Slomiany et al., 2009b; Sidhu et al., 2010; Wu et al., 2011). A first-line experimental testing of such a concept should seek to determine whether blood-borne merozoites or soluble forms of *Pf*Rh5 can indeed bind CD147 on KSHV-infected cells and whether those interactions are directly associated with a CD147-dependent alteration in cellular behavior, including extracellular remodeling and induction of a pro-angiogenic phenotype that is one of the defining features of the infectious process of KSHV (Qin et al., 2010; Cheng et al., 2011; Dai et al., 2012a,b; Gasperini et al., 2012). In **Figure 5**, we highlight some of the important nodes of intersection between KSHV and the malaria parasite in endothelial cells and skin-derived melanoma cells, along with their potential impact on the virus life cycle (i.e., reactivation), KS tumorigenesis, and malaria disease outcomes.

## THERAPEUTIC CONSIDERATIONS FOR KS AND MALARIA: CHALLENGES AND NEW OPPORTUNITIES

Kaposi’s sarcoma is one of the most frequent neoplasia diagnosed in malaria-endemic regions of Africa, yet despite recent progress (Casper, 2006; Casper and Wald, 2007), the landscape of effective therapeutic strategies for KS remains limited. It is clear that KS presents in different epidemiologic and clinical forms dictated by a variety of modifying risk factors, but the lack of data on the relative contributions of these co-factors in any given epidemiological setting frustrates efforts aimed at developing new approaches for clinical management of the disease. Surgical removal of isolated nodular KS does not eliminate latent KSHV at secondary sites, while traditional chemotherapy is generally toxic and may have a high failure rate in HIV-infected patients in whom durable post-therapy immune reconstitution is improbable. For epidemic (HIV/AIDS-associated) KS, intervention with the highly active anti-retroviral therapy (HAART) in HIV/HHV8 (human herpes virus 8) co-infected patients may have contributed to regression of KS lesions, but the initial success of HAART-based therapy for KS has been eroded by several concerns. First, HIV is not necessary for endemic African or classical Mediterranean KS. Second, HAART only limits the potentiating immunosuppressive effects of HIV but it does not remove the underlying etiology of KS. Third, HAART may reduce HIV viral load but it does not restore the entire T cell repertoire necessary for immunity against KSHV. Fourth, lack of access to HAART, non-compliance with the treatment, failure to respond to treatment, and the development of drug-resistant strains of HIV confound the overall benefit of a strictly HAART-based approach to KS management. Fifth, the benefits of HAART are not long-lasting and end up being more detrimental to many patients with advanced KS who may show no improvement while remaining in danger of developing post-therapy immune crisis (Krown et al., 2004). Sixth, the probability of recrudescence of KS in patients treated with HAART later in life is unpredictable. There is, therefore, an urgent need for a multi-pronged therapeutic approach aimed at developing strategies that are appropriate to the prevailing epidemiologic state of the disease, with the overall goal being improvement of the treatment outcome for KS patients in sub-Saharan Africa and other resource-limited parts of the world (McAllister et al., 2005).

The concept of “angio-therapy” designed to inhibit growth of angio-proliferative cancers like KS (Tosetti et al., 2002a,b; Ferrari et al., 2003; Pfeffer et al., 2003) led to the surprising observation that the anti-malarial peptide artesunate has anti-angiogenic effects on KS-derived endothelial cell lines (Dell’Eva et al., 2004). Artesunate is already well tolerated as an anti-malarial drug, and because it has direct effects against transformed cells, the promise of its dual use for KS is attractive for co-infected individuals. If adopted as such, the clinical benefits of artesunate for treatment of KS would represent a classic illustration of one of the defining attributes of a syndemic relationship in which the overall clinical impact of two linked infections (i.e., *Pf* and KSHV in this case) can be blunted by targeting the molecular underpinnings that link the parasite with the disease-modifying influence of KSHV. In addition, it has been proposed that quinine and its chloroquine and hydroxychloroquine derivatives – drugs that have been used widely to treat malaria – are immunosuppressive and may, as such, serve as co-factors for KS by stimulating KSHV reactivation, which would not only promote virus dissemination but could also support KS histogenesis (Ruocco et al., 2011). However, the “oncodrug” hypothesis for KS is likely to be more relevant for individuals with severely altered immunity since, in immunocompetent hosts, the viremic state induced by quinine and its derivative drugs would concomitantly expose hematogenously disseminating virions to immune surveillance which could in turn limit virus spread.

With respect to malaria control, approaches that interrupt the parasite life cycle are ideal, yet in spite of many multi-national efforts in this regard, successful elimination of the disease remains a major challenge, as more than half of the world’s population still lives in areas where there is a risk of contracting the disease. There are many reasons for this sobering report card, chief among them being persistent endemicity as a result of drug and insecticide resistance, inadequate support for malaria control programs, poor environmental management, the complex biology of the disease, as well as the regional variability not only in the parasite but also in the nature of its impact on specific populations and age groups. Another major challenge remains the lack of practical and affordable animal models that can faithfully reflect mechanisms of malaria pathogenesis and immunity in humans. Such platforms would be valuable for evaluating the efficacy of drug and vaccine candidates, and for predicting the benefits of drug combinations that can maximize safety and efficacy while minimizing the development of drug resistance.

Given that the invasive asexual blood stage of the *Pf* lifecycle is the form associated with symptomatic malaria, recent efforts toward a malaria vaccine have primarily focused on targeting this stage. In this respect, the discovery of the essential merozoite invasion receptor increases the number of potential targets for a second-generation malaria-specific vaccine based on CD147/*Pf*Rh5 interactions. However, the fact that deletion of other *Pf*Rh proteins also impairs invasion, albeit in a strain-specific manner, complicates vaccine design efforts, as it suggests that the *Pf*Rh5/CD147 interaction may be only one of many ligand–receptor recognition events that must occur during execution of the invasion process (Cowman and Crabb, 2006; Tanne, 2011; Tham et al., 2012). An approach that targets CD147 may not be feasible, as it could impact many important physiological processes controlled by this molecule. On the other hand, innovations oriented toward development of vaccine and therapeutic strategies based on the invariant aspects of *Pf*Rh5 and other “accessible” *Pf* antigens may result in a more meaningful outcome associated with minimal impact on the human host. Such strategies may include the combined use of nanovehicle-deliverable peptide mimetics and single-chain antibodies that can be administered before or during active parasitemia, or therapeutic lentiviral vectors carrying immunogenic epitopes that can harness the host’s immune capacity. These pathogen-centered approaches could be used alone or in conjunction with the RTS,S vaccine that is based on the most prominent surface antigen of the pre-liver sporozoite stage and which has already shown some promise in phase III trials (Tanne, 2011). In using *Pf*Rh5 as the target, however, the primary goal of any given approach [antibody-based (Douglas et al., 2011), or otherwise] would be to elicit the safest, most long-lasting and most efficacious outcome that limits the availability of *Pf*Rh5 to mediate invasion, but it must also be guided by the recognition that this antigen is predominantly located within the rhoptries and it is liberated and or revealed to the immune system only for a short period of time when the merozoite contacts the erythrocyte prior to invasion.

## CONCLUSIONS AND FUTURE PERSPECTIVES

Infectious agents have long been implicated in the etiology of a variety of illnesses, and although recent studies have examined the role of microbial co-infections in many disease settings, it is not known whether “cooperative pathogenesis” is sufficient to provide the driving force behind strategies in which co-pathogenic agents co-evolve and forge a state of forbearance with each other and with their shared host to advance mutually exclusive existential goals. Clearly, *Pf* malaria has been linked to a variety of other infections that display co-pathogenic relationships with the parasite, notable among them being eBL (Thorley-Lawson and Allday, 2008), but recent advances have revealed that many other important examples of these relationships do exist, and in most cases they provide a surprisingly informative conceptual window into how co-infections can modify each other’s disease course. For instance, the recent elucidation of the molecular mechanisms of erythrocyte invasion and cyto-adherence has: (a) illustrated the extent to which *Pf* can exert its impact on human physiology; (b) exposed how alterations in the expression and function of the host receptors that support these processes could not only dramatically change the dynamics of malaria but may also influence the pathogenesis of other co-infections such as KSHV that exploit these pathways for existential benefit; and (c) generated new interest in the structural dispositions of these receptor/ligand pairs as potential multi-domain vaccine targets against specific stages of the *Pf* life cycle.

Although malaria is not considered a typical opportunistic infection in the same way that KSHV is, recent advances have revealed a surprising node of intersection between KSHV and malaria pathogenesis at the level of the molecular controls that regulate the persistence of these two highly successful infectious agents. To the extent that such interactions can be measured at a micro level, the concept of co-pathogenesis establishes grounds for the expectation that KSHV and *Pf* can bidirectionally influence the clinical course of each other at many physiological levels leading to a variety of clinical outcomes (**Table 1**; **Figure 5**). For example, CD36-mediated cyto-adherence, by virtue of its ability to stimulate pathways that overlap with those required for KSHV reactivation, may contribute to a transient increase in KSHV viral load and dissemination, which in turn could increase the frequency of other defining correlates of KSHV-associated disease that rely on a viremic state. On the other hand, the pathogenic mechanisms of a latent KSHV infection, which include KSHV-induced downregulation of CD36, could effectively alter the overall disease burden by limiting peripheral sequestration; this could in turn increase parasitic access to the central nervous system, leading to a higher probability for cerebral malaria. Ultimately, the emerging picture supports a syndemic link which, however serendipitous, reveals a co-evolutionary paradigm centered at the putative dueling role of CD36 as a mediator of parasite sequestration on one hand and KSHV replication on the other. In this regard, more extensive molecular and genetic analysis is required in order to determine the extent to which KSHV (or EBV, for that matter) might provide the driving force for altering the genetic registry at the CD36 locus in regions of high malaria endemicity. It might also be necessary to analyze the CD36 locus in hematopoietic versus peripheral B cells that may serve as the vehicle for virus dissemination *in vivo*, in order to determine whether infection increases the propensity for a heritable lesion at this locus.

**Table 1:** Summary of the effects of KSHV on CD36 and CD147 expression and signaling, and examples of their potential impact on KSHV and *P. falciparum* disease pathogenesis.

Cell type	Target molecule	Effect of KSHV	Potential impact on KSHV pathogenesis	Potential impact on *P. falciparum*
Endothelial cells (HMVEC, t-D/MBEC, and LEC)	CD147	Up	• Increased angiogenesis through VEGF	• Increased trans-signaling by soluble *Pf*Rh5
			• Increased membrane metalloprotease (MMP) activity promotes tumor metastasis
	CD36	Down	• Associated with establishment of latency	• Reduced peripheral sequestration
	TSP-1	Down	• Promotes the angiogenic phenotype	• Enhanced probability for cerebral malaria
Skin-derived (Melanoma) cells (Mel1700 and Sk-Mel28)	CD147	Up	• Indeterminate	• Increase in merozoite invasion and parasitemia
	CD36	Up	• The increase in binding of *Pf*EMP-1 to upregulated CD36 leads to virus reactivation	• Increase in CD36/*Pf*EMP-1 binding may lead to enhanced sequestration of parasitized erythrocytes
	TSP-1	Up	• Angiostatic effects of TSP-1 are associated withvirus reactivation

*Endothelial cells tested are: HMVEC, human microvascular endothelial cells; t-D/MBEC, immortalized mixed dermal/microvascular brain endothelial cells; and LEC, Lymphatic endothelial cells. Skin (melanoma)-derived cells tested are Mel1700 and Sk-Mel28 (ATCC). TSP-1, Thrombospondin-1.*

In conclusion, recent advances have revealed several nodes of pathobiological intersection between malaria and a variety of clinically significant infections, and although substantial progress has been made, we are still in the “embryonic stage” of understanding how co-infections interact with each other in their mutual host. Derivative new research emphasis that is inspired by these concepts should include the important goal of developing practical *in vivo* platforms (animal models) that could facilitate systematic, experimental integration of population studies with reductionist multi-component molecular data. Although we are still a long way from developing such a platform for studying the malaria and KSHV co-infection paradigm, attempts toward this goal are an essential step in elucidating the extent to which triangular interactions between *Pf*, KSHV, and their mutual human host might articulate a syndemic relationship that underlies the co-incidence of aggressive KS in parts of the world with endemic malaria. Once the correlates of co-pathogenesis are isolated, innovative research efforts oriented toward development of effective “combined” therapies can be launched.

## Conflict of Interest Statement

The authors declare that the research was conducted in the absence of any commercial or financial relationships that could be construed as a potential conflict of interest.
