# Erratum: Dangerous liaisons: molecular basis for a syndemic relationship between Kaposi's sarcoma and *P. falciparum* malaria

**DOI:** 10.3389/fmicb.2013.00281

**Published:** 2013-09-19

**Authors:** Katelyn L. Conant, Anita Marinelli, Johnan A. R. Kaleeba

**Affiliations:** Department of Microbiology and Immunology, Uniformed Services University of the Health SciencesBethesda, MD, USA

**Keywords:** Kaposi's sarcoma, HHV-8, malaria, PfEMP-1, CD36, basigin/CD147, PfRh5, erratum

The authors wish to advise our readers that Anita Marinelli was omitted from the original author list in error. The correct list and order of authors should be: Katelyn L. Conant, Anita Marinelli, and Johnan A. R. Kaleeba. All authors support this change and heartily apologize for any inconvenience that the error may have caused.

